# Targeting Indoleamine 2,3-Dioxygenase 1: Fighting Cancers *via* Dormancy Regulation

**DOI:** 10.3389/fimmu.2021.725204

**Published:** 2021-09-03

**Authors:** Chao Yang, Chan-Tat Ng, Dan Li, Lei Zhang

**Affiliations:** ^1^National Engineering Research Center For Marine Aquaculture, Institute of Innovation & Application, Zhejiang Ocean University, Zhoushan, China; ^2^Department of Psychology, National Chengchi University, Taipei, Taiwan; ^3^Department of English, National Chengchi University, Taipei, Taiwan; ^4^State Key Laboratory of Southwestern Chinese Medicine Resources, School of Pharmacy, Chengdu University of Traditional Chinese Medicine, Chengdu, China; ^5^Sericultural Research Institute, College of Biotechnology, Jiangsu University of Science and Technology, Zhenjiang, China; ^6^Department of Chemical Engineering, Waterloo Institute for Nanotechnology, University of Waterloo, Waterloo, ON, Canada

**Keywords:** tumour dormancy, immunosuppression, kynurenine, nanotechnology, IDO1 regulation, aryl hydrocarbon receptor

## Abstract

The connection between indoleamine 2,3-dioxygenase 1 (IDO1) and tumour dormancy – a quiescent state of tumour cells which has been consistently linked to metastasis and cancer recurrence – is rarely discussed despite the pivotal role of IDO1 in cancer development and progression. Whilst the underlying mechanisms of IDO1-mediated dormancy are elusive, we summarize the IDO1 pathways which potentially contribute to dormancy in this review. Critically, distinct IDO1 activities are involved in dormancy initiation and maintenance; factors outside the well-studied IDO1/kynurenine/aryl hydrocarbon receptor axis, including the mammalian target of rapamycin and general control nonderepressible 2, appear to be implicated in dormancy. We also discuss various strategies for cancer treatment *via* regulating IDO1-dependent dormancy and suggest the application of nanotechnology to deliver effective treatment.

## Introduction

Cancer cells often remain unaltered and hidden until appropriate conditions for further progression are met. This status can be explained by tumour dormancy which is defined as a stage during which dormant cancer cells reversibly enter a G0 phase of the cell cycle and exhibit neither proliferative nor apoptotic characteristics; at the population level, it is characterised as a quiescent state of equilibrium in which a balance is maintained between tumour cell division and death (1, 2). Dormancy appears to be a critical stage for cancer development since it promotes the long-term survival of tumour cells: dormant tumour cells are barely detected by the immune system, eventually contributing to metastases, cancer recurrence, or tolerance to anti-cancer treatments (3–5). Since tumour dormancy poses a challenge in fighting against cancers, uncovering its underlying mechanisms is essential.

Previous literature has extensively investigated factors associated with the dormancy mechanisms, and indoleamine 2,3-dioxygenase 1 (IDO1) has been identified as one of the players [for a comprehensive review of molecular cues involved in tumour dormancy, see (6)]. IDO1 acts as a rate-limiting enzyme in the kynurenine biosynthetic pathway. By converting tryptophan (Trp) into kynurenine (Kyn) in peripheral tissues, IDO1 triggers subsequent immunoregulatory events, including the suppression of T cells and NK cells and the activation of regulatory T cells and myeloid-derived suppressor cells (7, 8). Although the IDO1 expression in most normal adult tissues is low, it is highly increased in a wide range of human cancers (9, 10). Being regulated by interferons (IFNs), overexpressed IDO1 suppresses apoptosis and promotes tumour dormancy in tumour-repopulating cells (TRCs), a highly tumorigenic subpopulation of undifferentiated tumour cells (11–13). Furthermore, blockade of the IDO1 pathway disrupts the dormancy state and induces TRC apoptosis (11, 12). IDO1 inhibition has, therefore, been proposed as a potential strategy to break dormancy, promote tumour suppression, and simultaneously improve other anti-cancer treatments, including chemotherapy.

Despite that IDO1 blockers have shown encouraging effects towards tumour suppression in preclinical settings and phase I/II trials, they are far from satisfactory in phase III clinical development [focused reviews on the development of IDO1 inhibitors are available (9, 14, 15)]. In particular, treatment of melanoma with the use of Epacadostat (a selective inhibitor of IDO1) combined with pembrolizumab (an inhibitor of PD-1) does not offer significant advantages over placebo plus pembrolizumab (16). This finding implies a gap in the current literature regarding the role of IDO1 pathways in cancer development. Moreover, whilst a large body of literature had focused on eliminating active cancers through IDO1 inhibition, investigation on the relation between IDO1 and tumour dormancy is lacking. We herein review the current efforts on investigating the interactions between IDO1 signalling and tumour dormancy and discuss possible directions for future research on cancer therapy *via* targeting these interactions.

## Upstream Regulation of IDO1-Associated Dormancy

**Figure 1:**
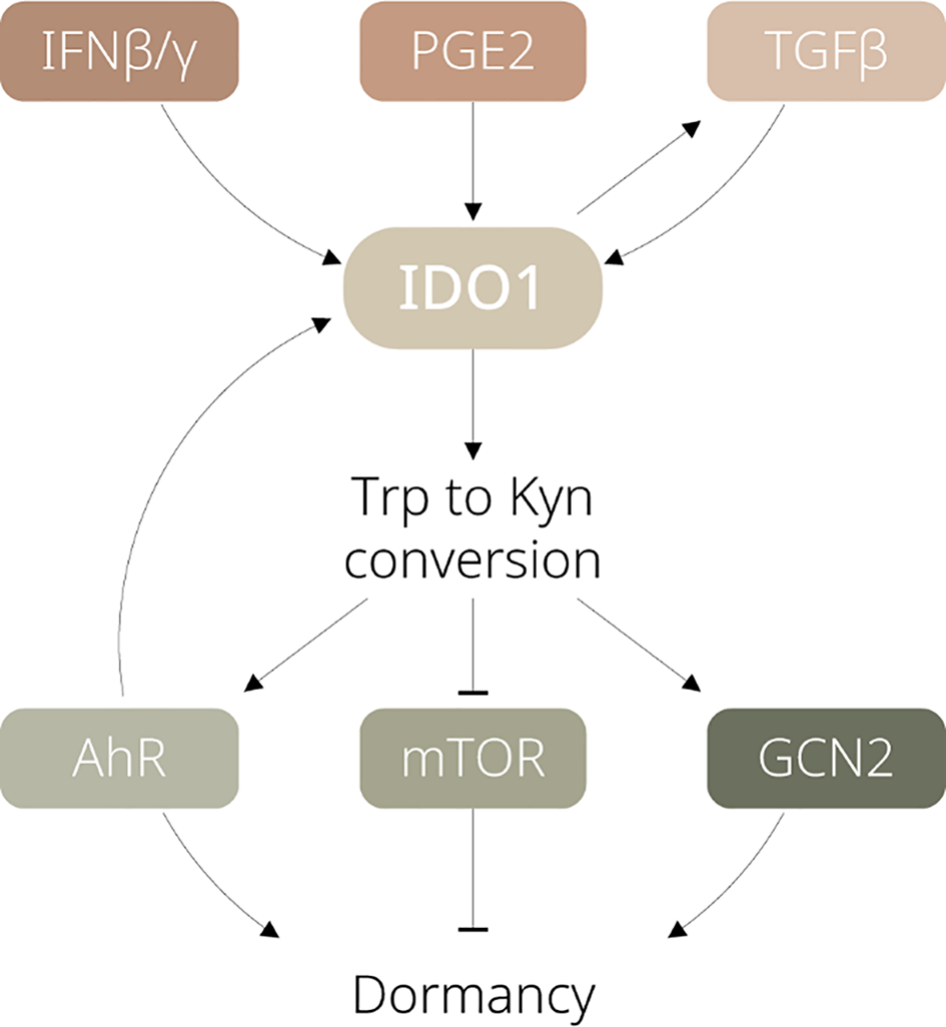
Schematic diagram illustrating the signalling pathways involved in IDO1-dependent tumour dormancy. IFN, interferon; PGE2, prostaglandin E2; TGF, transforming growth factor; Trp, tryptophan; Kyn, kynurenine; AhR, aryl hydrocarbon receptor; mTOR, mammalian target of rapamycin; GCN2, general control nonderepressible 2.

Major factors contributing to IDO1 activation and regulation have been well documented, including interferons, tumour necrosis factor-alpha, pathogen- and damage-associated molecular patterns *via* JAK/STAT, NF-κB, or other signalling pathways (10). However, how exactly these factors induce or maintain tumour dormancy remains to be clarified. In this section, we summarise the upstream pathways of IDO1 which could be critical for regulating dormancy (see **Figure 1**).

### Interferons Trigger Dormancy in Tumour-Repopulating Cells

Interferons (IFNs) are signalling proteins expressed, primarily by cytotoxic T lymphocytes, NK cells, and plasmacytoid dendritic cells (pDCs), in response to viral infection (17). They can trigger the JAK/STAT signalling pathways and elevate the expression of interferon-stimulated genes. These genes promote apoptosis of the infected cells and are involved in further immune responses. Since tumour cells and virus-infected cells tend to activate similar responses, the IFN pathways also improve the anti-cancer responses by inducing tumour cell apoptosis.

**Figure 2:**
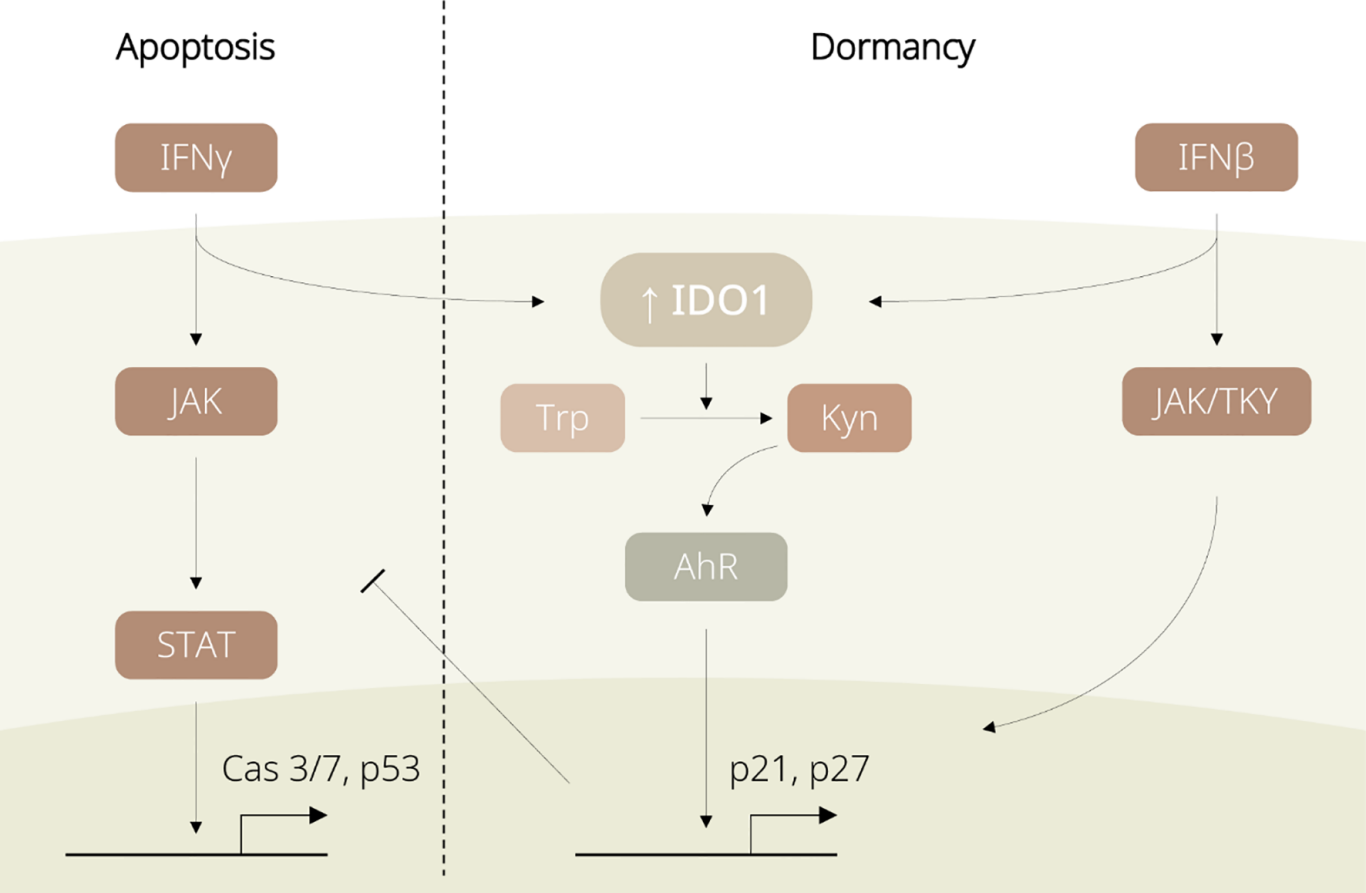
Diagram displaying regulation of IDO1-associated dormancy by IFN family members. IFN-γ induces apoptosis in non-tumour repopulating cells (non-TRCs) *via* the JAK/STAT pathway. In TRCs, IFNs favour the IDO1/Kyn/AhR pathway and promote p27 expression, leading to cell cycle arrest and tumour dormancy.

However, stem-like TRCs are capable of hijacking such mechanisms for protection against the immune system (11, 12). Through the IDO1/Kyn/aryl hydrocarbon receptor (AhR) metabolic cascade, IFN-γ upregulates p27 to suppress the JAK/STAT signalling and simultaneously induces G0/G1 cell cycle arrest in TRCs (11). In addition, activated IFN-β is also reported to lead to TRC dormancy, by either initiating Jak1/Tyk signalling for IDO1-independent p27 upregulation, or activating IDO1 and sharing the IDO1/Kyn/AhR metabolic circuitry with IFN-γ signalling (see **Figure 2**) (12). Being able to induce IDO1-mediated immunosuppressive events and tumour dormancy, IFNs are considered as a key upstream player of IDO1 for switching TRCs into the dormancy state.

The IFN-stimulated IDO1/Kyn/AhR cascade is favoured over the JAK/STAT pathway in TRCs rather than in differentiated tumour cells. This difference between tumour subpopulations might be explained by their distinct expression profiles that TRCs exhibit higher levels of IDO1 and AhR than differentiated tumour cells do (11, 12). Even though IDO1 expression is temporarily enhanced by IFNs in non-TRCs, the levels of IDO1 and AhR are likely insufficient to trigger the downstream IDO1 signalling. Further, IDO1 inhibition has been evidenced to be associated with suppression of the IDO/Kyn/AhR/p27 axis, thereby driving the dormancy-to-apoptosis transition. In other words, the initially high levels of IDO1 could govern the domination of the IFN-induced IDO1/Kyn/AhR circuitry and determine the entry to dormancy; blockade of the IFN/IDO1 signalling potentially disrupts the dormancy state.

Additionally, SOX2, which acts upstream of IDO1 and downstream of IFN-γ, is identified as another potential key involved in TRC dormancy (18). As a well-known biomarker for stem-like cells, SOX2 is upregulated in a wide range of cancers to support tumour proliferation and metastasis, and elevated SOX2 levels are associated with drug resistance (19–22). However, suppressed SOX2 appears to be required for IFN-stimulated TRC dormancy (18). A similar conclusion is reached by another study which has indicated IFN-γ increases IDO1 and inhibits SOX2 expression in glioma stem cells (23). It is rational to slow down proliferation triggered by SOX2 to promote tumour dormancy, yet how these factors interact with each other during dormancy is unclear. Future studies should explore the relations among SOX2, the IFN/IDO1 cascade, and tumour dormancy.

Whilst the positive correlation of activities between IDO1 and IFN-γ is strong in colorectal cancers, for example, it is relatively weak in ovarian or kidney cancers such that they may show great IDO1 expression but less activity of IFN-γ. Thus, researchers should consider the variation of IDO1 and IFN-γ activities in different cancer cell lines, because the inconsistent relations between these two factors have implied (1) the presence of other IDO1 inducers and (2) multiple stages of dormancy captured (9, 10). Importantly, the IDO1 regulation in dormant cells is supposed to change over time, but their temporal profile is rarely dissected. Usually, at the early stages of tumour progression, the production of immunosuppressive factors by tumours is poor, allowing high levels of IFN secreted by the CTLs or NK cells for IDO1/AhR activation. Nonetheless, later stages are associated with stronger suppression of T cells or NK cells, and thus the subsequent IFN levels might be lowered. Indeed, several studies concerning the IDO1/Kyn/AhR metabolic axis argue that IFN-γ is needed only for initiation but not maintenance of tumour dormancy – some other factors have been involved in the consecutive IDO1 activity (24–26).

Collectively, IFN signalling appears to be crucial for initiating tumour dormancy. Yet, distinct mechanisms have been revealed, at least, between the initiation and maintenance of tumour dormancy, or between different tumour cells. One should pay special attention when attempting to alter the dormancy state through IDO pathway regulation. Merely targeting the IFN pathways seems to be insufficient for effectively controlling the IDO-dependent tumour dormancy.

### Constitutive Expression of IDO1 Induced by Cyclooxygenase-2 Derived Prostaglandin E2

For the maintenance of tumour dormancy, the mechanical characteristics in the local tumour microenvironment have exerted important functions. For example, not only can stiffness facilitate p21 and p27 expression to trigger TRC dormancy, but it also helps maintain the dormancy state *via* inhibition of integrin β3 (27). In the meantime, stiffness affects the expressions of IDO1 as well as cyclooxygenase-2 (COX-2) in mesenchymal stem cells: both of their expressions are elevated even without cytokine stimulation (including IFNs) (28), suggesting that cytokines might not be necessary for IDO1 upregulation. More importantly, these results reveal a potential relationship between IDO1 and COX-2 under stiff conditions – a mechanical environment directly associated with TRC dormancy.

COX-2 and its downstream effector Prostaglandin E2 (PGE2) are now considered as potential contributors to cancer development and IDO1-dependent tumour dormancy (29, 30). COX-2 stimulated PGE2 facilitates IDO1 production *via* the PI3K/AKT and PKC signalling (30–32). Therefore, modification in any site within these pathways potentially results in the constitutive expression of IDO1. Expectedly, overexpression of COX-2/PGE2 is common in a broad range of human cancers, and it has been implicated in the mechanisms of anti-cell death and immunosuppression through Bcl-2 upregulation and IL-12 downregulation (33–38). Unlike IFNs, the COX-2 derived PGE2 allows consecutive IDO1 activity, and thus it appears to be responsible for the maintenance of the dormancy state. Consistently, the PGE2 pathways are reported to mediate the shift from dormancy to tumour growth, and suppression of these pathways awakes dormant tumour cells and sensitises them to immunotherapy (30, 39–42).

Apparently, the PGE2 signalling serves a crucial role in balancing apoptosis and growth in tumours, at least partly, through IDO1 mediation. Future research should examine how the interplay between IDO1 and PGE2 may impact on the initiation and maintenance of tumour dormancy. We also anticipate a systematic investigation of different cancer cell lines which should guarantee a more advanced understanding of the underpinnings of tumour dormancy.

### Transforming Growth Factor-Stimulated Sustained Expression of IDO1

Aiming at the cancer-associated events triggered by IDO1 pathways, most researchers focused on the enzymatic effects of IDO1. Nonetheless, previous literature has demonstrated the noncatalytic activity of IDO1, particularly in plasmacytoid DCs, for induction of immunosuppression (43–45). Specifically, TGF-β1 treatment in pDCs in mice induces phosphatidylinositol-3-OH kinase (PI3K)-dependent phosphorylation of the IDO immunoreceptor tyrosine-based inhibitory motifs (ITIM1/2). The phosphorylated IDO1 further recruits the tyrosine phosphatases SHP1/2 and activates the non-canonical NF-κB pathway, eventually leading to an upregulated expression of TGF-β1 and IDO1. Moreover, the IDO1-dependent NF-κB pathway is not interrupted by 1-methyl-tryptophan (1-MT), a blocker of the IDO1 enzymatic activity, indicating that the noncatalytic IDO1 signalling could drive the self-sustained feedback loop of IDO1 (45).

In line with these studies, TGF-β promotes immunoregulatory events, including the differentiation of the regulatory T cells (Tregs) (46, 47). At the same time, TGF-β1-induced IDO1 signalling in pDCs upregulates IFN-β, a potential facilitator of tumour dormancy (45). On the other hand, IDO-competent DCs are likely to be recruited to induce IDO1 at a low level of IFN-γ in a rat model (24). Although IFN-γ drives a rapid immunoregulatory effect, it is independent of the long-term activation of IDO1; stable IDO1 activity is instead maintained in the IDO+ DCs by the IDO1 enzymatic activity as well as the increased AhR for a feedback IDO1-AhR-IDO1 loop in a self-sustained manner (25). Moreover, TGF-β1 expression is downregulated by IFN-γ in the model of Li and colleagues (25), suggesting a sequential activation of IFN-γ and TGF-β1. These findings indicate that the IDO1-associated dormancy could be maintained independently of the IFNs. However, how the high level of IFN-stimulated IDO1 expression in tumours is related to the IDO1 levels in DCs during dormancy remains to be explored. The intercellular crosstalk between the TGF and IFN pathways requires further investigation.

## Downstream Mechanisms of Dormancy Driven by IDO1-Induced Tryptophan-to-Kynurenine Conversion

### Aryl Hydrocarbon Receptor-Dependent Immunosuppression

As discussed in the previous section, the AhR signalling is activated by the IDO1-mediated conversion of kynurenine from tryptophan (11, 12). The activated AhR up-regulates p21 and p27 expression (48, 49), further contributing to cell-cycle inhibition and immunosuppression. Notably, in IFN-stimulated TRCs, the preference to the IDO1/Kyn/AhR cascade requires not only a high concentration of IDO1 but also the activated AhR, indicating the essential role of AhR in TRC dormancy (11). Consistently, the AhR binding with its ligand kynurenine results in Treg differentiation (50). It is marked that TGF-β activation elevates the expression of AhR, and optimisation of the TGF-β-driven Treg differentiation mostly relies on AhR (50–52). Given the profound relations between IDO1 and TGF-β, and between IDO1 and the downstream AhR, it will be of interest to examine whether the TGF-β-AhR interaction is directly mediated by IDO1. Regardless, AhR likely acts as an important effector of IDO1 and contributes to the TRC dormancy entry.

### Suppression of Mammalian Target of Rapamycin Results in T-Cell Anergy

Other than the IDO1/Kyn/AhR signalling, IDO1-mediated immunosuppression can also be achieved through the mammalian target of rapamycin (mTOR) signalling (53, 54). The mTOR pathway is involved in cell cycle control (55). Additionally, mTOR activation through the regulation of PI3K/AKT signalling is essential for the functional activity of cytotoxic T cells. IDO-mediated degradation of tryptophan deactivates the mTOR signalling, thereby expanding Foxp3+ Tregs and suppressing cytotoxic T cells (56, 57). Previous studies have indicated that tryptophan degradation boosts differentiation of Tregs, and the subsequent conversion of kynurenine further leads to T-cell apoptosis (58, 59). It is noted that IFN stimulation does not immediately cause IDO1-induced intracellular depletion of tryptophan in dormant TRCs, which probably is achieved by the increased tryptophan transporters (11, 60). Therefore, impaired mTOR expression due to the extracellular deprivation of tryptophan at the local site can be explained by the enhanced tryptophan uptake of the dormant TRCs. Also, inhibition of IDO1 turns the tryptophan deficiency signal into the sufficiency signal, and the sufficient supply of tryptophan reactivates mTOR and relieves the dormancy state (54). As a result, the tryptophan-sensitive mTOR signalling serves as a potential effector pathway of IDO1, regulating the T immunity for immunosuppressive events including dormancy.

### General Control Nonderepressible 2 Activation Leads to T-Cell Anergy

The general control nonderepressible 2 (GCN2) is identified as another sensor of tryptophan sufficiency, and always works together with the mTOR pathways to regulate T cell activity (61, 62). Whilst mTOR signalling is impaired in response to tryptophan deprivation, IDO1-mediated degradation of tryptophan stimulates the GCN2 pathway, eventually diminishing T-cell proliferation and enhancing apoptosis (59, 63). However, the role of GCN2 in T-cell depletion and immune tolerance is lately questioned. Particularly, in a melanoma mouse model, the IDO-triggered immune responses towards tumours were unaltered regardless of the presence of GCN2 (64). Yet, it is also demonstrated that GCN2 is needed for survival in previously activated T cells, but incapable of controlling the proliferation of naive T cells (53). Whether GCN2 is redundant or otherwise specialised with additional functions regarding dormancy is still unclear.

## Prospective in Targeting IDO1-Associated Dormancy as Cancer Therapy

### Breaking the Dormant State of Tumours Through IDO1 Inhibition

Owing to the critical role in tumour-associated immunosuppression, IDO1 appears to be a potential target for cancer treatment (54, 65–70). For example, 1-methyl-tryptophan (1MT) is widely used as an inhibitor for the IDO1 pathway in preclinical studies, although its validity is questioned. Later research has further suggested indoximod, the D racemer of 1MT, as a potential candidate in the clinical settings. Indoximod can attack cancer cells by restoring the suppression of mTOR signalling caused by tryptophan deprivation, thereby acting on T cells. However, it should be noted that indoximod does not target directly at IDO1, but disrupts the downstream Kyn pathways in an unknown way. In addition, Epacadostat, an IDO1 blocker through competitive inhibition, has been shown to enhance the growth of T and NK cells and suppress the differentiation of Tregs, although the phase III study of Epacadostat was not satisfying (9, 13). The negative results did not stop the investigations of IDO1 inhibitors, but have called for more efforts to their development as well as understanding the underlying mechanisms (71–73).

Van den Eynde et al. have summarised possible reasons and corresponding solutions specifically for the unsatisfactory clinical trial of Epacadostat (9). For example, Epacadostat tends to show the best inhibition against cancer cells with constitutive expression of IDO1 (i.e., the cancer cells produce IDO1 even when they are not inflamed) rather than those with inducible activity (i.e., the cancer cells produce IDO1 in response to T-cell infiltration); the concentration of Epacadostat administered may be insufficient for blocking the IDO1/AhR pathway; as mentioned earlier, there are multiple sources of IDO activation at different stages; IDO1 expression varies among different cancer cell lines. Whilst many of the inhibitors, including Epacadostat, are designed against the enzymatic activities of IDO1, few act on its non-catalytic functions. All these factors might contribute to the overall survival not being improved in the clinical trial of Long et al. (16). Critically, understanding why IDO1 inhibitors failed in clinical trials may aid the future development of IDO-based cancer treatment.

Targeting IDO1-associated dormancy yet lacks practicality, and few IDO inhibitors are tested against tumour dormancy. We expect future research to work on the impacts driven by IDO1 regulation, particularly on the dormancy mechanisms in addition to the active tumours. This should be crucial for the success of IDO1 treatment development, as tumour dormancy has been strongly associated with metastasis and later resistance to cancer treatments (74, 75). It should also be noted that there are other enzymes, including tryptophan-2,3-dioxygenase and IDO2, which are closely related to IDO1, yet they could have complementary or differential roles during immunosuppression: TDO triggers the same Kyn/AhR downstream signalling with IDO1 that they show nearly identical bioactivity (76); IDO2 is documented to implement in the IDO1-dependent regulation of T cells (77), but it is also reported that IDO1 and IDO2 may show opposite roles in immune responses (78). Although TDO and IDO2 are not the current focus of this article, researchers should pay close attention to them as targeting tumour dormancy *via* the Kyn/AhR signalling may not always be IDO1 specific.

### Stabilising IDO1-Dependent Dormancy for Tumour Suppression

Perhaps paradoxically but intriguingly, it is wondered whether dormancy promotion is always pessimistic, or it may serve as an alternative strategy for controlling cancers. Dormant tumour cells are notorious for their hardly being detected or treated, but they have limited abilities to grow and proliferate. Promoting or stabilising dormant tumour cells, as a consequence, might slow down cancer progression and thereby provide alternative therapeutic effects. Moreover, previous mathematical models suggest that dormancy can be theoretically maintained for a long period of time, if not permanently (79). This paradigm is proposed as tumour dormancy therapy: instead of aiming at tumour elimination *via* aggressive treatments, the pro-dormancy approaches may suppress tumour cell growth and enhance cancer patients’ quality of life in a gentler manner (80–82). Tumour cells tend to remain dormant when low-dose immune checkpoint inhibitors are applied with hyperthermia [reviewed by Corthay and coworkers (83)]. Although being insufficient for eliminating cancers, low-dose inhibition prevents tumour cells from further progression. As an immune checkpoint enzyme, IDO1 is considered to be an appealing target for such a therapeutic approach.

Recently, chemotherapy itself has been identified to induce tumour dormancy, and the relation between chemotherapy and dormancy could be bridged by the type I IFN signalling (84). This suggests that we may even proactively activate the dormancy mechanisms to suppress tumour growth *via* the IDO-related pathways. Nonetheless, some studies suggest tumour cells may become more difficult to re-enter dormancy once they escape from immunosurveillance, plausibly because of the aggravated mutation rates (85–87). Researchers should consider how and when this paradigm should be applied, or otherwise it could be deteriorative not to provide sufficient suppression.

**Figure 3:**
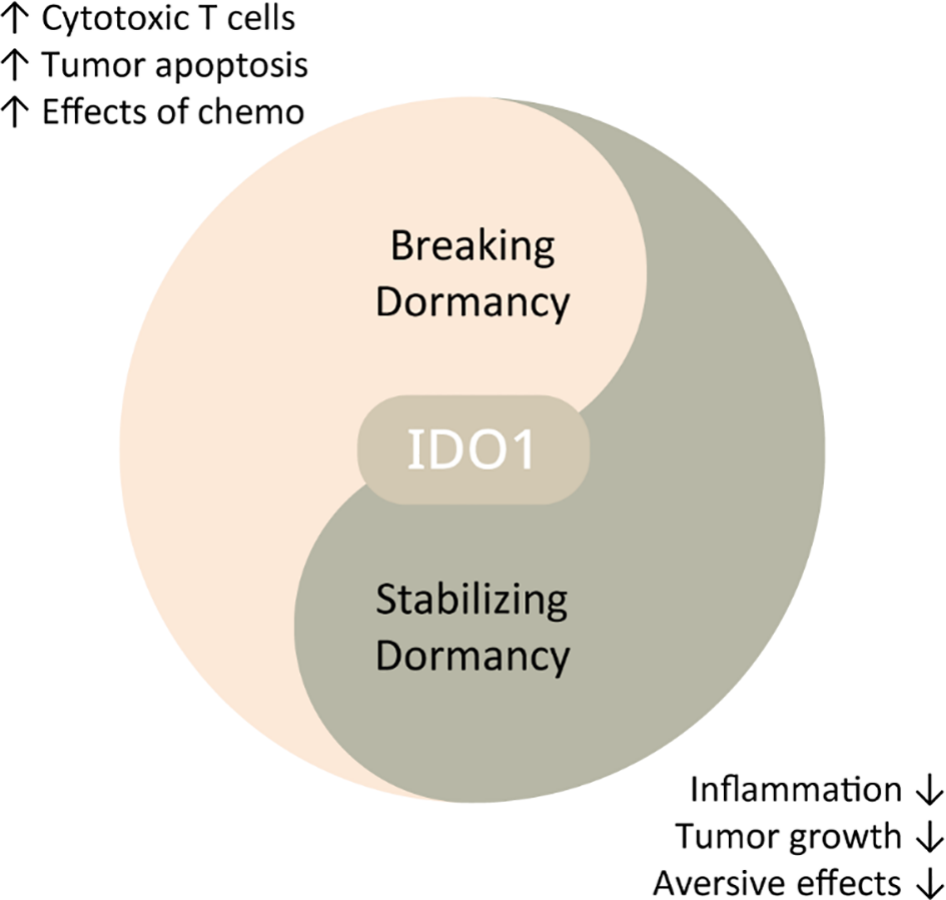
Schematic diagram displaying potential therapeutic strategies against cancers through IDO1 regulation. IDO1 inhibition may break dormancy, thereby promoting immune surveillance and enhancing other anti-cancer therapies. Low-dose IDO1 suppression can induce dormancy, alleviating tumour growth and tumour-related aversive effects.

To emphasise, we are not advocating the replacement of conventional strategies which aim to break or prevent dormancy; however, tumour dormancy therapy can be used in concert with these established tumour-killing methods at different stages to achieve optimal outcomes (see **Figure 3**). Yet, no studies to date have attempted to bring forward the idea of promoting or stabilising dormancy *via* regulating IDO1 as a potential strategy to control cancer progression.

## Nanomaterials as a Delivery Platform of IDO1

How we should deliver treatments to the appropriate sites of the targeted cell types with controlled release is another challenge. Unsuccessful treatment delivery not only diminishes the therapeutic effects, but also intensifies the side effects. Tackling the issue, emerging nanomaterials are able to provide more flexible platforms for the delivery of small-molecule inhibitors, thereby improving cancer therapy (88–90). The application of nanotechnology could even solve some potential causes of the unsuccessful Epacadostat clinical trial and is briefly discussed in the following.

Currently, IDO1 antagonists combined with nanotechnology have demonstrated encouraging outcomes against tumorous events. For instance, PEG-Fmoc-NLG has been proposed as a dual-functional nanocarrier such that the carrier itself can improve immune responses and simultaneously be able to deliver both chemotherapy and IDO1 inhibition in mouse models of melanoma and breast cancer (91). More recent efforts have resulted in the modified polyvinyl alcohol (PVA)-based nanogels being identified to deliver IDO1 inhibitors together with chemo-medicines in a mouse breast cancer model. This delivery system has shown specificity of drug release at intracellular acidic conditions, and has led to the cytotoxic effects against the tumour cells and the reduction of IDO1-associated immunosuppression (92). Other nano-enabled approaches for a combination of IDO1 downregulation and chemotherapy may include the cationic lipid-assisted nanoparticles (CLANs) and DOX/IND-liposomal delivery systems (93, 94).

Based on these findings, we may anticipant nanotechnology to aid the development of IDO1 inhibitors and provide solutions for the failure of Epacadostat clinical trial. For example, the maximum allowable doses of inhibitors are usually restrained due to the trade-off between beneficial and adverse effects. As a result, the allowed administered level of IDO1 blockers may be insufficient to halt the IDO1/AhR signalling (9). Nanotechnology allows precise delivery that minimises unwanted effects and increases the allowed doses.

On the other hand, the use of nanotechnology has also been evidenced to be effective for controlling tumour dormancy. In an orthotopic mice model of colorectal tumour, injection of a lipid-protamine-DNA (LPD) nanoparticle loaded with PD-L1 trap synergises with chemotherapy (95). Such a combination therapy delivers precise treatment and therefore reduces adverse effects. More importantly, it can sensitise the immunologically cold tumour cells to the treatment. Liu et al. has recently developed a tumour-microenvironment (TME) sensitive nanocarrier for co-delivery of mitoxantrone and celestrol with an optimal ratio, leading to a successful prolongation of the dormancy state in desmoplastic melanoma (96). Not only have these findings validated the use of nanomaterials for breaking dormancy with accurate treatment delivery, but also they have pioneered the pro-dormancy strategy using nanomaterials. It would be exciting to examine whether these nanotechnology-based delivery systems are also useful for controlling IDO1-associated dormancy.

## Conclusion

Previous literature has suggested IDO1 should be connected to immunosuppression and tumour dormancy. However, there is still much room for understanding how exactly these IDO1 pathways result in dormancy. One of the keys falls on the IDO1 expression patterns, and constitutive rather than simply inducible IDO1 expressions may be critical for entering the dormancy state. Furthermore, initiation and maintenance of dormancy appear to involve distinct mechanisms such that they require different molecular factors to participate. Having a more thorough understanding of these mechanisms should provide novel insights into dormancy regulation and cancer treatment. Regarding the perspective on IDO1-centered dormancy regulation, while current research has devoted to the development of IDO1 inhibitors for active tumour elimination, how these inhibitors may be applied in dormancy models needs further attention. On the other hand, pro-dormancy therapy through IDO1 activation may serve as an alternative strategy for cancer treatment, yet it requires additional investigation in the future. Finally, given the potential of nanotechnology, we foresee an ever-rising use of nanomaterials for providing accurate delivery of treatments targeting IDO1-associated dormancy and enhancing cancer therapy.

## Author Contributions

CY and C-TN conceived and designed this manuscript. DL and ZL have read, revised and approved the manuscript. All authors contributed to the article and approved the submitted version.

## Funding

The work was supported by the Natural Science Foundation of Jiangsu Province, China (Grant No. BK20190960), the Natural Science Foundation of the Jiangsu Higher Education Institutions of China (Grant No. 19KJB180014), China Postdoctoral Science Foundation (2019M663456 and 2019TQ0044), Xinglin Scholar Research Promotion Project of Chengdu University of TCM (BSH2019008), Sichuan Province Science and technology innovation seedling project (2020091), Open Research Fund of Chengdu University of Traditional Chinese Medicine Key Laboratory of Systematic Research of Distinctive Chinese Medicine Resources in Southwest China (2020BSH004) and Canada Mitacs Fellowship (IT18262).

## Conflict of Interest

The authors declare that the research was conducted in the absence of any commercial or financial relationships that could be construed as a potential conflict of interest.

## Publisher’s Note

All claims expressed in this article are solely those of the authors and do not necessarily represent those of their affiliated organizations, or those of the publisher, the editors and the reviewers. Any product that may be evaluated in this article, or claim that may be made by its manufacturer, is not guaranteed or endorsed by the publisher.
